# Mobile Mental Health Screening in EmotiZen via the Novel Brain-Inspired MCoG-LDPSNet

**DOI:** 10.3390/biomimetics10090563

**Published:** 2025-08-23

**Authors:** Christos Bormpotsis, Maria Anagnostouli, Mohamed Sedky, Eleni Jelastopulu, Asma Patel

**Affiliations:** 1 Department of Artificial Intelligence and Computational Neuroscience, EmotiZen GmbH, 55122 Mainz, Germany; a.patel122@aston.ac.uk; 2 Research Immunogenetics Laboratory, First Department of Neurology, School of Medicine, National and Kapodistrian University of Athens, Aeginition University Hospital, Vas. Sofias 72-74, 11528 Athens, Greece; managnost@med.uoa.gr; 3 Multiple Sclerosis and Demyelinating Diseases Unit, Center of Expertise for Rare Demyelinating and Autoimmune Diseases of CNS, First Department of Neurology, School of Medicine, National and Kapodistrian University of Athens, Aeginition University Hospital, 11528 Athens, Greece; 4 Department of Computing, School of Digital, Technology, Innovation and Business (DTIB), The University of Staffordshire, College Road, Stoke-on-Trent ST4 2DE, UK; mhs2@staffs.ac.uk; 5 Department of Public Health, School of Medicine, University of Patras, 26504 Patras, Greece; jelasto@upatras.gr; 6 Department of The Operations and Information Management, Aston Business School, Aston University, Birmingham B4 7ET, UK

**Keywords:** brain-inspired models, deep learning, mobile health applications, digital mental health, artificial intelligence

## Abstract

Anxiety and depression affect millions worldwide, yet stigma and long wait times often delay access to care. Mobile mental health apps can decrease these barriers by offering on-demand screening and support. Nevertheless, many machine and deep learning methods used in such tools perform poorly under severe class imbalance, yielding biased, poorly calibrated predictions. To address this challenge, this study proposes MCoG-LDPSNet, a brain-inspired model that combines dual, orthogonal encoding pathways with a novel Loss-Driven Parametric Swish (LDPS) activation. LDPS implements a neurobiologically motivated adaptive-gain mechanism via a learnable β parameter driven by calibration and confidence-aware loss signals that amplifies minority-class patterns while preserving overall reliability, enabling robust predictions under severe data imbalance. On a benchmark mental health corpus, MCoG-LDPSNet achieved AUROC = 0.9920 and G-mean = 0.9451, outperforming traditional baselines like GLMs, XGBoost, state-of-the-art deep models (CNN-BiLSTM-ATTN), and transformer-based approaches. After transfer learning to social media text, the MCoG-LDPSNet maintained a near-perfect AUROC of 0.9937. Integrated into the EmotiZen App with enhanced app features, MCoG-LDPSNet was associated with substantial symptom reductions (anxiety 28.2%; depression 42.1%). These findings indicate that MCoG-LDPSNet is an accurate, imbalance-aware solution suitable for scalable mobile screening of individuals for anxiety and depression.

## 1. Introduction

Mental conditions such as depression and anxiety burden millions of people around the world [1,2]. With the exponential increase in such mental illnesses, innovative solutions like online platforms and mobile applications (apps) for screening anxiety and depression have emerged, attempting to face them [3,4]. For example, online platforms such as Psycho Web have been developed to collect data from cases diagnosed with mental disorders. The Psycho Web platform utilizes the k-nearest neighbours (KNN) algorithm to identify the type of mental disorder a patient is grieving from based on patient symptoms when evaluated by a mental health professional [5].

Recently, techniques for predicting mental health conditions, primarily anxiety and depression, widely used in current mental health mobile and web apps, rely on classical statistical, machine techniques, notably Generalized Linear Mixed Models (GLMMs), Logistic Regression (LR), Support Vector Machines (SVM), Naïve Bayes (NB), Decision Trees (DT), Random Forests (RF), Adaptive and eXtreme Gradient Boosting (AdaBoost and XGBoost), and deep learning methods like neural networks, but without specifying the architecture of neural networks that employed [6,7,8]. However, due to the imbalanced nature of mental health datasets, machine and deep computational models face limitations, such as overfitting, when processing datasets where early clinical deterioration signals are weak compared to most non-critical data. This necessitates models that can adapt to rapidly changing patterns [9]. Such imbalances can bias predictive models and the majority class, reducing their sharpness to individuals who could benefit most from an early screening of their mental health. Even though these machine techniques have advanced our understanding of how mental health issues may occur, they often struggle with the complexities inherent in the psychological data of anxiety and depression, which limits their effectiveness and poses key challenges such as:

- Applying machine learning to mental health predictions requires greater caution and the development of innovative, domain-adapted methodological techniques [10,11].
- The machine and deep learning models exhibit limited generalization ability due to class imbalance in the datasets [12].
- There is limited research that can provide evidence of the effectiveness of mobile apps in mental health anticipation. The lack of a robust evaluation for computational models makes it challenging to confirm that the algorithms incorporated in mental health apps effectively achieve their intended purposes and deliver tangible benefits to users [6].

Based on the above-mentioned limitations of machine and deep learning approaches applied to mental health prediction, this study’s objective is to utilize techniques to improve computational models in the prognosis of mental issues, particularly anxiety and depression. Researchers have employed network improvement approaches to address challenges posed by imbalanced datasets, which heavily rely on ensemble learning [13]. However, it may be challenging for ensemble learning to handle the complexities of imbalanced data, as ensembling dense architectures can lead to overfitting due to inefficiencies in training and deployment [14].

Recent developments in neurofinance, an interdisciplinary approach that merges economics, neuroscience, and psychology, have culminated in the MCoRNNMCD-ANN, a biologically inspired architecture designed to tackle the challenges of imbalanced, high-dimensional time-series forecasting [15]. Drawing on the brain’s modular structure and capacity for synchronized yet orthogonal communication pathways, the MCoRNNMCD-ANN model maintains resilience against non-stationary, skewed data distributions and excels at detecting rare but high-impact events that often elude conventional computational approaches.

Likewise, by embedding principles of modularity and orthogonality, MCoRNNMCD-ANN boosts predictive accuracy within financial markets and demonstrates broad applicability across other complex systems characterised by intertwined biological and behavioural processes.

The MCoRNNMCD-ANN’s adoption of modularity and orthogonality has advanced predictive accuracy in neurofinance and highlighted the broader applicability of these principles to other complex biological and behavioural domains [16,17]. Notably, MCoRNNMCD-ANN has been recently praised as one of the leading cognitive models in business intelligence, where its ability to predict infrequent but consequential outcomes has been ascertained as invaluable in decision-support systems [18].

MCoRNNMCD-ANN’s flexible framework seamlessly integrates global pattern recognition with localized feature extraction. Furthermore, MCoRNNMCD-ANN’s combination with NLP components, such as word embeddings, could better capture unlabelled text sentiment data’s semantic and syntactic features. Capturing localized features and global behavioural shifts is important for accurate text classification and may enhance early mental detection [19]. A recent study has proposed a neuroscience-inspired AI framework that constructs cognitive models, such as MCoRNNMCD-ANN, in conjunction with NLP, thereby elevating predictive accuracy in neurofinance and extending its applications inherently into mental health [20].

Building on biological and neuroscience foundations, this study addresses the twin challenges of class imbalance and calibration in mental health detection, aiming to improve predictive power and generalizability for the early detection of anxiety and depression in mobile-health applications. We therefore propose the Modular Convolutional orthogonal Gated Loss-Driven Parametric Swish Network (MCoG-LDPSNet), a novel variant of MCoRNNMCD-ANN that contains two orthogonal gated recurrent subnetworks, one specialized for anxiety and one for depression, which learn disentangled, emotion-specific representations. This work makes a significant contribution by integrating these subnetworks with a single, loss-driven gain mechanism that is co-optimized with the learning objective. The subnetwork outputs are fused and immediately passed through a first-of-its-kind Loss-Driven Parametric Swish (LDPS) layer: LDPS implements a single learnable gain parameter (β) that dynamically modulates the fused activation. Two complementary loss drivers control β with distinct effective timescales: (i) a phasic driver (Focal Loss) produces large, sample-specific gradients on complex or minority examples and transiently up-regulates β, amplifying weak or underrepresented emotional cues (an effect inspired by acetylcholine transients that sharpen cortical responses) [21,22]; and (ii) a tonic driver (Brier-score regularizer) supplies a slower, aggregate gradient that dampens β when the network becomes overconfident, thereby improving calibration. In practice, β is initialized to a moderate value and hard-clipped during optimization to avoid runaway gain. The LDPS layer is trained together with upstream regularizers (dropout, SpatialDropout1D, orthogonally initialized GRUs) so that amplification is selective and robust rather than permissive of memorization [23]. This biologically inspired, dual-timescale modulation enables the network to boost minority cues when needed [24,25,26]. At the same time, preserving calibrated probability estimates is a balance we verify empirically, as presented in Section 3 and Section 4.

Moreover, this study evaluates MCoG-LDPSNet’s overall performance with a geometric mean (G-mean) and ROC analysis. These objective evaluation metrics are better choices when applied to imbalanced datasets for medical diagnosis and text classification [27]. This study utilizes a publicly available mental health corpus for anxiety and depression on Kaggle (https://www.kaggle.com/code/mesutssmn/sentiment-analysis-for-mental-health/input, last accessed on 30 May 2025), benchmarked against classic statistics such as LR and GLMM, machine learning algorithms like RF and XGBoost, state-of-the-art deep learning models like DeprMVM and CNN-BiLSTM-ATTN (CBA), and transformer models like BERT. Furthermore, this study employs transfer learning fine-tuning of MCoG-LDPSNet on Facebook, which includes anxiety and depression data from Islam et al. [28], to leverage broader linguistic representations while adapting to domain-specific, anxiety- and depression symptomatology-informed visualizations for users. Correspondingly, a cohort study is conducted to evaluate the proposed MCoG-LDPSNet performance integrated into the EmotiZen (https://emotizen.health/, last accessed on 30 May 2025) mobile and web app, which delivers continuous, on-demand screening for anxiety and depression.

This study addresses two principal questions:

- Detection Efficacy: Does MCoG-LDPSNet substantially improve the early detection of anxiety and depression under severe class imbalance compared to traditional and state-of-the-art machine and deep learning approaches?
- Mobile Feasibility: How does integrating the proposed MCoG-LDPSNet model improve the EmotiZen App’s accuracy, scalability, and personalization in detecting early signs and predicting symptom severity of anxiety and depression in real-time?

The contributions of this study can be expressed as follows:

- First-in-Domain Bio-Inspired Dual-Path Text Model: This study introduces MCoG-LDPSNet, the first cognitive framework to apply bio-inspired MCoRNNMCD-ANN neurofinance principles to mental health text classification. By deploying parallel encoders for anxiety and depression, each with orthogonal-initialized GRUs, SpatialDropout1D regularisation, and SReLU gating, the proposed model disentangles affective cues and delivers significantly higher accuracy than both machine baselines and leading transformer-based approaches under severe class imbalance. Section 4 presents a detailed comparison and discusses whether the novel MCoG-LDPSNet is better suited to generalize in the imbalanced mental health dataset.
- Novel Loss-Driven Parametric Swish (LDPS) Activation: To the best of the authors’ knowledge, no prior model has directly fused phasic Focal Loss and tonic Brier score within a Parametric Swish activation to drive its gain dynamics. In the proposed MCoG-LDPSNet, the learnable gain β is rapidly up-regulated by Focal Loss, emphasizing hard, minority-class examples, inspired by transient neuromodulatory bursts, and gently down-regulated by Brier score regularisation, enforcing well-calibrated, steady confidence, akin to tonic neuromodulation. This unique phasic–tonic dual-loss coupling, implemented within a single activation layer and augmented by sparsity constraints, uniquely equips the proposed MCoG-LDPSNet model to sharpen its sensitivity to rare emotional signals while suppressing false negatives. Section 3, Section 4 and Section 5 investigate whether the new LDPS activation is in place to enhance the performance of the MCoG-LDPSNet further.
- Real-Time Adaptive Screening: Integrated within the EmotiZen mobile app, MCoG-LDPSNet updates its predictions in real time as new user inputs arrive. This real-time adaptability enables early, accurate screening of anxiety and depression symptoms, facilitating timely intervention. Section 5 discusses the real-time adaptability of the proposed MCoG-LDPSNet, which could enhance the early screening of anxiety and depression in the EmotiZen App.

The proposed MCoG-LDPSNet operationalizes biomimetic principles by mapping neural activities to model components. The architecture employs parallel, orthogonally initialized dual encoders for anxiety and depression, introducing a novel LDPS activation that couples a phasic, focal-loss driven gain boost with a tonic, Brier-score based calibration signal to adapt the Parametric Swish gain during training. These biomimetic-inspired design choices increase sensitivity to rare emotional signals, mitigate severe class imbalance, and enable a deployable on-device pipeline for real-time screening in the EmotiZen App, demonstrating how nature-informed engineering can meet practical challenges in digital mental health.

The rest of this paper is organized as follows:

Section 2 reviews the neuroscience foundations for neural networks, as well as state-of-the-art machine and deep learning models for predicting anxiety and depression. Section 3 illustrates and emphasizes the proposed architecture of the MCoG-LDPSNet model, estimating its effectiveness. Section 4 presents the results from a detailed comparative analysis of the proposed MCoG-LDPSNet model against both traditional and cutting-edge models from the literature, along with a discussion of these findings. Section 5 examines the practical implications of deploying the proposed MCoG-LDPSNet model in the EmotiZen App, as well as its potential for more accurate screening of anxiety and depression. Section 6 wraps up the principal findings of this research, addresses its limitations, and suggests directions for future work.

## 2. Neuro-Inspired Deep Learning for Early Detection of Anxiety and Depression

This study thoroughly examined multidisciplinary fields, including artificial intelligence, informatics, mental health, neuroscience, neurobiology, and traditional and state-of-the-art machine and deep learning approaches. The primary objective is to comprehensively synthesize existing conceptual and empirical articles, encompassing both secondary and primary research, through a meta-narrative review [29]. A semi-systematic review has also proven sufficient to gain a better understanding of complex areas, such as sentiment and natural language processing research [30,31,32]. To maximize predictive performance and generalizability, the development of the proposed MCoG-LDPSNet model followed a multi-stage learning and validation pipeline. Initially, MCoG-LDPSNet was trained on a large mental health corpus from Kaggle (https://www.kaggle.com/code/mesutssmn/sentiment-analysis-for-mental-health/input, last accessed on 30 May 2025) to learn broad representations of emotional and linguistic patterns relevant to anxiety and depression. The MCoG-LDPSNet was benchmarked quantitively against conventional and state-of-the-art machine learning and deep learning models, determining which approaches most effectively predict mental health outcomes related to depression and anxiety. Subsequently, the proposed MCoG-LDPSNet model underwent transfer learning using the Islam et al. Facebook dataset [28], allowing the MCoG-LDPSNet to refine its parameters and adapt to the nuances of social media discourse linked to anxiety and depression. The transfer learning strategy leveraged the strengths of large-scale and domain-specific data, resulting in a robust MCoG-LDPSNet model capable of nuanced mental health prediction.

The proposed MCoG-LDPSNet model was then integrated into the EmotiZen App for real-world deployment and evaluation. EmotiZen GmbH conducted a cohort study to validate the effectiveness of the proposed MCoG-LDPSNet. Primary data were collected from two cohorts of EmotiZen App users: one group used the standard version of EmotiZen, which offered weekly mental health recommendations and screenings, while the other engaged with an enhanced version featuring additional user engagement tools, such as a progress bar in connection with recommendation selections. During the study period from 1 January 2025 to 31 March 2025, longitudinal data on anxiety and depression symptoms were collected directly through the app. The predictive accuracy and practical relevance of the proposed MCoG-LDPSNet were assessed using this primary cohort dataset, allowing for rigorous evaluation of both the proposed model’s performance and the EmotiZen App’s impact on mental health outcomes in real-world settings. This end-to-end design, from large-scale pretraining and transfer learning to real-world cohort validation, demonstrates the practical utility and translational potential of the proposed approach for digital mental health support, as detailed in Section 4. User consent and protocol were established for the primary data collection from EmotiZen GmbH to ensure the accuracy of the results and to ensure that the ethics of the app are fully applied in compliance with regulations regarding data privacy, thereby ensuring the ethical conduct of this study, as outlined in the Declaration of Helsinki.

The initial screening in this study yielded 1250 research papers from Scopus (*n* = 850), IEEE Xplore (*n* = 300), and Web of Science (*n* = 100), encompassing studies from a broad range of years to ensure historical and contemporary coverage. After manually duplicating all records, 1064 records remained. An exhaustive review, using inclusion and exclusion criteria, observing strategically titles, abstracts, and keywords to determine the investigations most likely to be of interest to this research, resulted in 120 records being reviewed. A final selection of 35 studies was made, obtaining the most relevant and high-quality evidence for this analysis.

The inclusion criteria mandated that studies must meet the following requirements:

- Be English-language publications in peer-reviewed, reputable journals or conference proceedings;
- Focus on predictive models for screening or prognosis of mental health conditions, particularly anxiety and depression;
- Validate their predictive performance using quantitative objective evaluation metrics such as AUROC, precision, recall, or F1-score;
- Use state-of-the-art computational techniques, including traditional machine learning methods (e.g., LR, DT, RF), advanced deep learning architectures (e.g., CNN-BiLSTM-ATTN (CBA), and transformer-based models like BERT);
- Contribute to developing or enhancing bio-inspired, neuroscience-informed or cognitive models relevant to the brain.

The exclusion criteria included excluding studies that met the following requirements:

- Were published in non-English-language, which may have limited methodological innovation;
- Were not peer-reviewed or empirically validated;
- Lacked quantitative or experimental rigour and did not incorporate robust evaluation measures;
- Focused exclusively on unrelated domains.

Key keywords that emerged from this study literature included the following:

“Mental Health”, “ Predictive Models”, “Anxiety”, “Depression”, “Imbalanced Datasets”, “Bio-inspired Models”, “Cognitive Architectures”, “Modular Neural Networks”, “Brain Processes”, “Sentiment Analysis”, “Machine Learning”, “Deep Learning”, “Transfer Learning”, “Ensemble learning”, “Logistic Regression”, “Support Vector Machines”, “Naïve Bayes”, “Decision Trees”, “Random Forests”, “eXtreme Gradient Boosting”, “CNN”, “RNN”, “BERT”, and “Mobile Mental Health Applications”.

**Figure 1:**
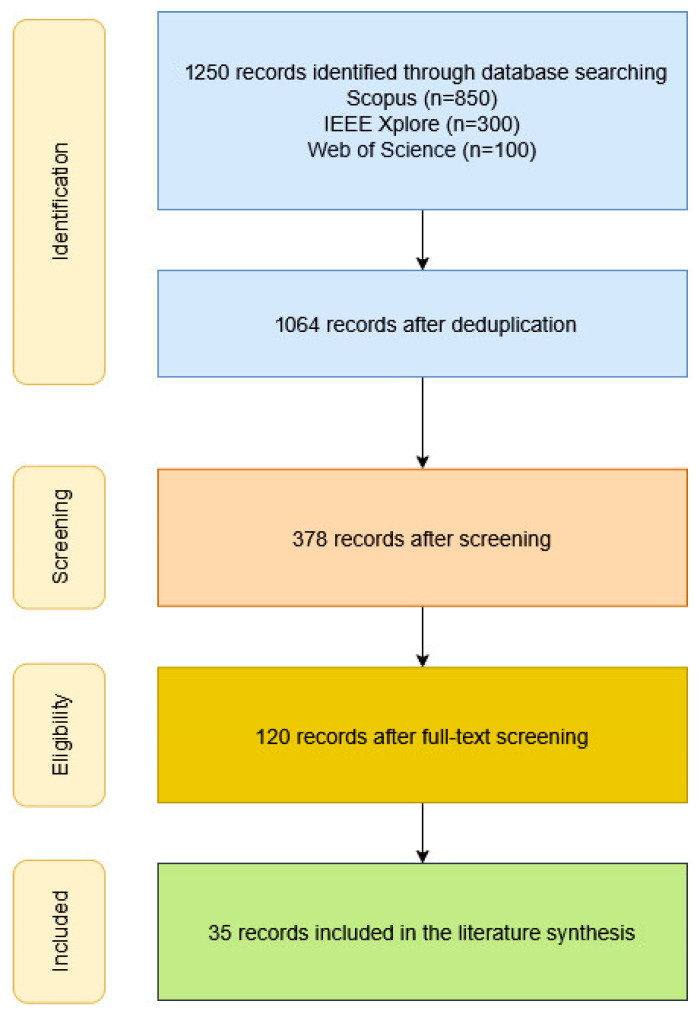
The flowchart documents justifications from the data extraction and quality assessment.

Figure 1 illustrates the study selection process that was followed.

To structure this study’s literature synthesis, neuroscience foundations are considered to examine biological insights on brain modularity, vmPFC, anterior insula, amygdala circuitry, and inhibitory interneuron pathways that inform the design of resilient, predictive models. The following computational models cover the evolution from traditional statistical and machine-learning classifiers (e.g., LR and RF) through deep learning architectures (CNNs and RNNs), transformer-based methods (BERT), and hybrid state-of-the-art systems (e.g., CNN-BiLSTM-ATTN and DeprMVM). Prior methods have made noteworthy refinements in feature extraction and sequence modelling; a key novelty of this work lies in incorporating brain-inspired mechanisms into the MCoG-LDPSNet model’s architecture and learning dynamics. Specifically, the proposed MCoG-LDPSNet introduces a novel loss-driven adaptive activation function whose gain parameter is modulated by both focal loss and calibration regularization, thereby inspired by the dual timescale neuromodulatory processes observed in the brain. This biologically grounded design enables the proposed model to dynamically adjust its sensitivity to minority-class emotional cues and maintain robust calibration by addressing the challenges of class imbalance and overconfidence that persist in existing state-of-the-art methods. Thus, the proposed MCoG-LDPSNet represents a novel step beyond conventional architectures by incorporating neurobiological principles at the core of its predictive framework for early screening of anxiety and depression.

### 2.1. Biology and Neuroscience Foundations

One must first trace their roots back to neural substrates to elucidate how biologically inspired computational models can excel at predicting mental health outcomes. Early investigations combined neuroimaging, neuropsychiatric assessments, and brain stimulation studies to pinpoint depressive loci, implicating the prefrontal cortex, limbic structures, basal ganglia, and brainstem nuclei and revealing altered connectivity among these regions [33]. Complementary lesion and psychiatric analyses further underscored the amygdala, hippocampus, and thalamus as both primary and secondary hubs of depressive pathology, highlighting the need for distributed frameworks to capture complex affective processes [33]. Building on this foundation, researchers examined the regulatory influence of the ventromedial prefrontal cortex (vmPFC) over the amygdala in humans, specifically in relation to mood and anxiety. Their work tested a neurocircuitry model positing that vmPFC hypoactivity disinhibits the amygdala, elevating negative affect. Indeed, vmPFC lesions corresponded with heightened amygdala responses to aversive stimuli and increased resting-state amygdala connectivity compared to those of healthy controls, thereby cementing the vmPFC’s role as a key modulator of emotional reactivity and a potential therapeutic target [34].

Further studies delineated the inhibitory microcircuits that temper its output under anxiogenic conditions including specific GABAergic neuron subtypes in the basolateral and central nuclei and molecular determinants like gamma-aminobutyric acid (GABA), a neurotransmitter and chemical messenger in the brain. GABA receptors and synaptic organizer proteins were shown to gate anxiety responses, suggesting that fine-tuning inhibitory synapses could yield novel interventions for anxiety disorders [35]. Shifting to generalized anxiety disorder (GAD), functional MRI analyses revealed hyperactivation of the amygdala, vmPFC, and ventrolateral PFC during emotion regulation tasks. At the same time, resting-state scans exposed disrupted amygdala coupling with prefrontal, insular, and cerebellar regions. These findings framed GAD as a network-level disorder marked by emotional and cognitive dysregulation, warranting studies of at-risk populations to isolate its underlying neurobiology [36].

Parallel morphometric work across major depressive disorder (MDD), GAD, and panic disorder highlighted common and distinct cortical alterations within the prefrontal-limbic circuitry encompassing the amygdala, anterior cingulate, and prefrontal cortices and called for deeper exploration of frontotemporal and parietal contributions to these conditions [37]. Concurrently, advancements in systems neuroscience painted cognitive flexibility and resilience as emergent properties of a modular brain architecture, dynamically regulated by neuromodulatory signals like acetylcholine and dopamine that adjust cortical gain across the ventromedial and dorsolateral PFC. Disruptions in these modulatory processes have been linked to depression’s hallmark deficits in emotional regulation and cognitive control, offering blueprints for computational models to emulate the brain’s balance of sensitivity and stability, especially when tackling imbalanced mental health datasets [24,38,39,40,41].

Extending this integrative lens, recent investigations into Beck’s cognitive theory employed neuroimaging to map negative cognitive bias in MDD. Hyperactive amygdala responses foster fear and anxiety, hippocampal dysfunction skews memory toward harmful content, and PFC imbalances erode regulatory control together, perpetuating depressive thought patterns and guiding the development of bias-modification and targeted neuromodulatory therapies [42]. Against this neuroscientific backdrop, AI approaches have emerged to tackle diagnostic and prognostic challenges in psychiatry. Reviews of machine learning, such as SVMs, and deep learning, such as CNNs, demonstrated their potential for early detection and personalized treatment planning [43,44]. However, mechanistic models remain scarce and warrant further validation in adolescent and adult cohorts [45].

Bridging these domains, studies leveraging the Research Domain Criteria (RDoC) framework compared cognitive bias signatures across anxiety and depression, revealing both disorder-specific and transdiagnostic patterns that robustly predict symptom severity and point toward bias-informed cognitive interventions [46]. Collectively, these neurobiological insights serve as a scaffold for designing deep learning architectures, such as CNNs and RNNs, that can mimic brain-like modularity, connectivity, and neuromodulation to enhance the prediction of anxiety and depression. In the next section, we delve into the strengths of these machine and deep learning models, setting the stage for benchmarking with the proposed MCoG-LDPSNet’s brain-informed architecture.

### 2.2. Machine and Deep Learning in Mental Health

To predict mental health conditions such as anxiety and depression, reducing potentially the frequency and harshness of ongoing symptoms, researchers have proposed and applied several machine- and deep learning techniques. For example, researchers applied NLP and machine learning techniques to predict depression from text data on social media, comprising 1500 sentences gathered from platforms such as Facebook, Twitter, and Instagram. The researchers applied data preprocessing techniques, including tokenization, removal of stop words, removal of empty strings, removal of punctuation, stemming, and lemmatization. Six machine learning classifiers were used: Multinomial Naïve Bayes (MNB), LR, Linear SVC, KNN, RF, and DT. MNB and LR, achieving the highest accuracy compared to Linear SVC, outperformed it by 1.06%. MNB and LR performances were 2.12% better than RF, outperforming KNN by 4.30% and DT by 6.52%. These calculations indicate the superior performance of MNB and LR as they outperformed the other classifiers in all the comparisons. The researchers suggest that future research develop a mobile application incorporating machine learning to enable individuals to check their depression level [47].

Researchers utilized a CNN model to identify a user’s mental state based on social media posts. They aimed to detect whether users’ posts belonged to an exact mental disorder, including depression, anxiety, borderline, schizophrenia, and autism. For data, they collected posts from mental health communities on Reddit. The researchers considered that this model could help identify potential sufferers of mental illness based on their social media posts. NLP techniques were employed to tokenize the posts and filter out frequently used words, while XGBoost was operated for comparison with the CNN model. The CNN model outperformed XGBoost by 10.32%, indicating enhanced accuracy in identifying depressive symptoms. Similarly, in anxiety detection, the CNN model achieves a 9.98% higher accuracy compared to XGBoost, demonstrating improved performance in recognizing anxiety-related patterns. The consistent outperformance of CNN across both depression and anxiety detection suggests its more substantial generalization capabilities and effectiveness in handling the nuanced language patterns associated with mental health discussions on social media platforms. Finally, they proposed validating the CNN model with data from other social network services [48].

Researchers strived to manage the negative influence of the COVID-19 pandemic crisis on mental health, stressing that the early detection and intervention of depression prevent the illness from evolving to a more severe state and prevent the development of other health conditions. The study proposed a survey comprising 21 questions, based on the Hamilton tool and the advice of a psychiatrist, to collect data on depression. The data was then analyzed utilizing machine learning techniques like DT, KNN, and Gaussian Naïve Bayes (GNB). KNN provided better results in terms of accuracy, outperforming DT by 2.95% and GNB by 4.55%. Their study suggested using machine learning-based models to replace conventional methods of detecting sadness by asking people encouraging questions and obtaining regular feedback from them. For future research, they proposed further investigation into the use of machine learning in depression detection, as well as the exploration of other machine learning techniques and their effectiveness [49].

Researchers investigated anxiety in 127 university engineering students in India using machine learning, gathering data through a questionnaire that met the criteria for Likert scale measurement. Machine learning algorithms, including NB, DT, RF, and SVM, were applied to classify the anxiety level based on the consequences of anxiety after being trained on pre-existing questionnaire data points. The accuracy results revealed that RF emerged as the top-performing algorithm, surpassing the NB and DT by 10.50% and SVM by 4.40%. For future studies, they proposed focusing on implementing interventions based on the identified causes and effects of anxiety to support students’ mental health [50].

Likewise, researchers used a dataset of 61,619 college students from 133 US higher education institutions using machine learning predictive models to identify college students at heightened risk of anxiety and depressive disorders. Their study provided a practical tool for professional counsellors to identify at-risk students and proactively guide prevention and intervention strategies. Researchers utilized predictive benchmarks, including XGBoost, RF, DT, and LR. In terms of area under the curve (AUC), XGBoost demonstrated better performance in both anxiety and depression categories. For anxiety, XGBoost outperformed LR and RF by 1.37% and DT by 4.13%. In the depression category, XGBoost and RF tied at an AUC of 0.77, surpassing LR by 1.31% and DT by 5.33%. For future research, they proposed that the models be further validated and tested in different populations to assess their generalizability [51].

Researchers explored distinguishing symptoms between depression and anxiety and utilized a streamlined version of the Symptom Checklist 90 (SCL-90) with 4262 patients. To achieve this, they developed classification models, such as KNN, SVM, RF, and AdaBoost. The accuracy, AUC, precision, and F1 score were the objective metrics used to measure the SCL-90 outcomes by the classification models. Regarding AUC, SVM outperformed KNN by 1.38%, RF by 2.05%, and AdaBoost by 6.04%. Although SVM, RF, and AdaBoost achieved an accuracy of 94.38%, SVM surpassed KNN by 1.68%. Overall, SVM performed the best, especially in terms of AUC. For future research, it is suggested that researchers further test the generalizability of the classification models [52].

Recently, researchers developed a machine learning-based risk prediction model for depression in 2733 middle-aged and elderly individuals with hypertension in China, using a survey for data collected from the China Health and Retirement Longitudinal Study (CHARLS) between 2018 and 2020. Machine learning models, such as LR, RF, and XGBoost, were developed to compare their prediction efficiency in CHARLS. Comparing the AUC, the LR showed the highest AUC, outperforming XGBoost by 0.71% and RF by 1.71%. Researchers proposed that further research is needed to validate the findings in other samples [53].

A current study investigated the detection of depression on a dataset that includes structural (English) and non-structural (Roman Urdu) languages. Moreover, the datasets, one in Roman Urdu manually converted from English comments on Facebook and another in English from Kaggle, were merged for the experiments. The researchers compared the performance of various machine and deep models, including SVM, Support Vector Machine Radial Basis Function (SVM-RBF), RF, and BERT. The results show that SVM outperformed the SVM-RBF, RF, and BERT by approximately 2.41% in accuracy. The researchers recommended that future studies investigate advanced hybrid machine learning models to improve accuracy in predicting depression in European countries [54].

Researchers developed a predictive model for non-suicidal self-injury (NSSI) among adolescents in western China aimed to evaluate the risk of NSSI in Chinese adolescents using machine learning algorithm-based models. Their study collected sociodemographic and psychological data from 13,304 adolescents in 50 schools in western China. Their outcomes showed that the multivariate logistic regression (MLV) model identified several risk factors for adolescent NSSI, including gender, age, history of psychiatric consultation, stress, depression, anxiety, tolerance, and emotion abreaction. The XGBoost model identified depression and anxiety as the top two predictors of NSSI in adolescents. In the training set, XGBoost outperformed the MLV model in accuracy by 0.10%, but the MLV regression model had a higher AUC of 2.44%. XGBoost again demonstrated a slight increase in accuracy of 0.10% for the testing set, while the MLV regression model maintained a marginally better AUC of 0.36%. These slight discrepancies exhibited that both models perform very similarly, with XGBoost having a slight edge in accuracy and the logistic regression model showing a slight advantage in AUC. The overall predictive ability of both models appears to be strong and comparable, identifying several key predictors of NSSI, including depression and anxiety. They proposed that the models used could be further validated in other regions and populations for future research [55].

A recent study proposed an emotional and mental intelligence (EMI) chatbot for the early detection of mental health issues. The objective was to address the barriers of stigma, accessibility, and affordability in mental healthcare based on the notion of a Digital Twin, a virtual replica designed to represent a physical object in order to assess and classify mental health issues such as anxiety and depression. EMI was developed in collaboration with a clinical psychiatrist, and a pre-trained BERT model was employed to detect various severity levels. BERT detected symptoms of mental health with 69% accuracy. For future research, they recommended addressing the challenges of imbalanced datasets and focusing on the generalizability and scalability of their framework. Additionally, more comprehensive evaluation metrics and performance measures can provide a deeper understanding of the chatbot’s effectiveness in mental health assessment [56].

Depression has long been characterized as a pervasive mental health disorder, and the ubiquity of social media has opened new avenues for automated screening via text classification. A recent study suggested the usage of a Convolutional Neural Network in conjunction with a Bidirectional Long Short-Term Memory with attention mechanism CNN-BiLSTM-ATTN (CBA) model for depression detection. It benchmarked the performance of CNN-BiLSTM-ATTN (CBA) against seven established architectures: LSTM, BiLSTM, CNN, CNN-LSTM, CNN-BiLSTM, BiLSTM-Attention, and CNN-BiGRU, on the CLEF2017 dataset. Their proposed CNN-BiLSTM-ATTN (CBA) achieved an AUC-ROC of 0.85, outperforming LSTM/BiLSTM by 11.2%, CNN-BiGRU by 12.5%, BiLSTM-Attention by 7.32%, and the strongest CNN-based baselines by 3.6%. These results underscore the efficacy of the attentive hybrid architectures for more discriminative depression detection [57].

In a recent study, the researchers aimed to detect whether a person is depressed. SVM and multilayer perceptrons (MLP) were utilized to formulate an ensemble approach, namely, hybrid DeprMVM. A survey with psychological and sociodemographic features was used to collect data from 604 participants. They also operated data manipulation methods, such as SMOTE and cluster sampling, to improve accuracy. Their findings showed that the proposed ensemble of DeprMVM, which incorporates SMOTE and cluster sampling techniques, demonstrated notable improvements in AUC compared to other classifiers, outperforming KNN by 9.63%, SVM by 4.17%, and RFC by 2.06%. Compared to the high-performing XGB and MLP classifiers, the ensemble of DeprMVM still achieves a 1.03% improvement in both cases. They proposed that further research is needed to validate the effectiveness of their suggested ensemble approach in different populations and settings. They also proposed the development of a user-friendly tool based on the presented model that could be explored for practical applications in healthcare settings [58].

The studies mentioned above have achieved noteworthy results in predicting mental health conditions concerning anxiety and depression. However, researchers have pointed out that there is still significant potential for future improvement in the predictions of mental health issues. At the same time, significant opportunities remain to enhance model sensitivity, generalizability, and robustness, especially under imbalanced conditions in the real world. The gaps and future research suggestions can be summarized as follows:

- Researchers should create mobile applications incorporating AI subfields like machine learning to enable individuals to self-assess their depression levels [47].
- Researchers should investigate the deployment of user-friendly tools to support informed decision-making about mental health in real-world healthcare settings [58].
- Researchers should address the issues of imbalanced datasets and assess the framework’s scalability and robustness across diverse populations and settings [56].
- Researchers should investigate the effectiveness of different machine and deep learning algorithms in detecting mental health conditions, highlighting the need to improve model accuracy and generalizability [51,52,56].

Based on the above future directions and the limitations presented in Section 1, more research needs to be conducted in mental health to present new mobile applications that may support early screening and personal recommendation to individuals; moreover, the improvement of computational models’ accuracy and generalizability and their effectiveness in imbalanced datasets in mental health need to be addressed. Given these challenges, this study proposes the MCoG-LDPSNet, a novel neuroscience-informed framework extending the neurofinance-based MCoRNNMCD-ANN model. The proposed approach integrates a new brain-inspired LDPS mechanism to enhance further prediction of anxiety and depression in imbalanced datasets. The proposed MCoG-LDPSNet can also be incorporated into mobile tools, such as the EmotiZen App, for early and accurate screening of mental health. Ultimately, this integration could empower individuals with timely mental health insights for anxiety and depression signs, potentially enhancing well-being with internet-delivered cognitive behaviour therapy (iCBT) recommendations, decreasing the undiagnosed conditions [59,60,61].

## 3. Materials and Methods

### 3.1. Data Collection

The first dataset utilized from Kaggle contained anonymous statements with mental health statuses like normal, depression, suicidal, anxiety, stress, bipolar, and personality disorder, including most likely clean data. Moreover, the statements from Kaggle’s data (https://www.kaggle.com/code/mesutssmn/sentiment-analysis-for-mental-health/input, last accessed on 30 May 2025) encompassed diverse social media posts, and each entry was classified with a specific mental health status. This study utilized only anxiety, extracting 3888 statements, and depression, extracting 15,404 assertions. The data from Kaggle was deemed most likely clean and after initial checks, no further NLP cleaning techniques were performed for the text. It is worth noting that this study used a tokenizer, which filters out punctuation and converts text to lowercase by default, thereby reducing some noise. Further, deep learning approaches are more resilient and less sensitive to noise than traditional techniques.

The proposed MCoG-LDPSNet has been fine-tuned on domain-specific tasks, such as predicting anxiety and depression, using a smaller dataset from Islam et al. [28]. This dataset included a collection of Facebook page comments, totalling approximately 7000. Comments were labelled as anxiety if they contained anxiety and as depression if they contained depression. After this step, any comments that did not mention either mental condition were excluded from further analysis. The final dataset included 2446 comments labelled as anxiety and 337 as depression. This process confirmed that only comments relevant to this study of these two mental health states were included, resulting in a focused dataset for analysis.

Finally, this study collected primary data through a cohort study to validate the reliability of the proposed MCoG-LDPSNet, deployed in the EmotiZen App, for the prediction and early screening of anxiety and depression.

### 3.2. Proposed Model

**Figure 2:**
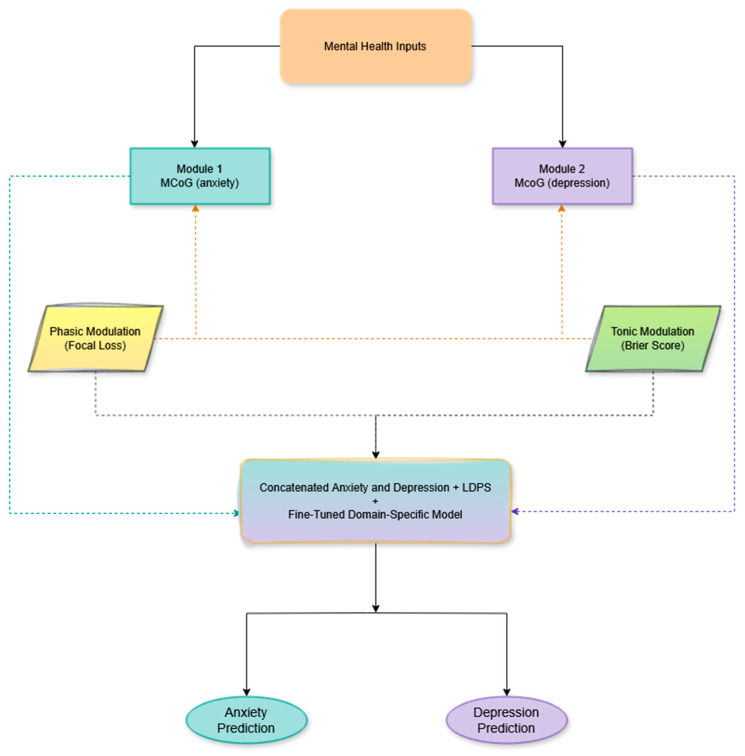
The proposed MCoG-LDPSNet comprises two modules for anxiety and depression (MCoG), with their outputs fused and passed to the LDPS, which incorporates Focal Loss inspired by transient neuromodulation and the Brier score for sustained calibration. Both modules pre-train knowledge fusion and fine-tune for domain-specific prediction of anxiety and depression.

The proposed MCoG-LDPSNet is a modular neural architecture motivated by the neurofinance MCoRNNMCD-ANN for binary classification, designed to disentangle anxiety and depression signals in text. It consists of two parallel text-encoder pathways, one for anxiety and one for depression. Each embedding tokens via a 64-dimensional SpatialDropout1D layer, extracting local n-gram features with Conv1D, pooling, and capturing sequence context through an orthogonally initialized GRU with PReLU. The final outputs of each module are concatenated into a joint representation, which is then passed through a learnable dense layer and the novel Loss-Driven Parametric Swish activation function before being output as a sigmoid. This novelty is achieved by embedding the LDPS layer whose gain parameter β is co-optimized by (1) Focal Loss, which down-weights easy negatives and amplifies gradients on occasional emotional cues, driving β higher for minority-class examples [21,22] and (2) Brier-score, which penalizes miscalibration during training to prevent β from overshooting [23]. This dynamic, error-driven nonlinearity is inspired by the brain’s adaptive processing and cortical gain control, which sharpens responses to unexpected stimuli [62]. By situating this LDPS layer immediately after fusion, the proposed network amplifies scarce emotional cues when they are most vulnerable to being overshadowed, yielding an imbalance-aware, intrinsically calibrated model. Figure 2 illustrates the proposed model for MCoG-LDPSNet.

#### 3.2.1. Module 1: Anxiety Text Encoder

The proposed MCoG-LDPSNet model operates on raw textual data on Kaggle representing user expressions or posts. Each input document is first pre-processed and tokenized to convert raw text into a numerical format suitable for neural processing.

The input text is segmented into a sequence of tokens (words, sub-words, or characters) denoted by the following:

$$x\in 1,\ldots ,V^{T},$$

V=10000;T=100

where T=100 is the fixed length of the token sequence per sample. Tokens beyond this length are truncated or padded. V=10,000 are the vocabulary entries, representing the most frequent tokens in the training corpus.

The anxiety module embeds tokens into a 64-dimensional space, applies spatial dropout, captures local patterns via 1D convolution, pools the features, and encodes temporal context using an orthogonal-initialized GRU with PReLU activation. Finally, a dense with SReLU layer produces the anxiety-specific vector $z_{a}$∈ ℝ^32^.

Each token $x_{t}$ is mapped to a dense vector representation, capturing semantic and syntactic properties:

$$E_{t}^{a}=W_{e}\;e\left(x_{t}\right),$$

- Here, $W_{e}\in R^{V\times d\;},\;d=64,$ is the embedding matrix, where d=64 is the embedding dimension.
- $e\left(x_{t}\right)$ is a one-hot vector for token $x_{t}.$
- The result is a sequence of embeddings with $Ea=\left(E_{1}^{a},\ldots ,E_{t}^{a}\right)E^{a}$, with $E_{t}^{a}$ $\in R^{64}.$

To reduce overfitting and encourage robustness, *SpatialDropout1D* with dropout probability p=0.2  is applied across embedding features:

$$E_{t}^{a}=\;\mathrm{SpatialDropout1D}\;(E_{t}^{a};p=0.2)$$

To detect local n-gram patterns, a 1D convolutional layer with kernel size 3 is applied across the sequence embeddings:

$$C_{t}^{a}=ReLU\;\left(E^{\prime a}*W_{c}+b_{c}\right),$$

where

$W_{c}\in R^{3\times d\times 64\;}\;$ is the convolutional filter.

The *ReLU* activation introduces non-linearity, enabling the model to learn complex feature representations.

Temporal max-pooling selects the most salient features across the entire sequence length:

$$h^{a\left(0\right)}=max_{t}C_{t}^{a},h^{a\left(0\right)}\in R^{64}$$

This yields a fixed-size summary vector, independent of sequence length, that emphasizes the strongest activations in each feature channel.

The pooled features are passed through a GRU recurrent layer, initialized with orthogonal weights to enhance gradient flow and stabilize training:

$$h_{t}^{a}=PReLU\;(W_{xh}^{a}h^{a\left(0\right)}+W_{hh}^{a}h_{t-1}^{a}+b_{h}^{a}),\;h_{t}^{a}\in R^{32}$$

The *PReLU* activation enables the network to adaptively learn the slope of the negative part, thereby further improving its expressiveness.

After temporal encoding, dropout regularization is applied:

$${\;h}^{\left(1\right)}\;\;=\;Dropout\;(h_{T}^{a}\;;p=0.3)$$

The final hidden state $h_{T}^{a}$ experiences dropout p=0.3 before being projected to the anxiety-specific embedding:

$$z_{a}=SReLU\;\left(W_{d}^{a}h^{\left(1\right)}+b_{d}^{a}\right),z_{a}\in R^{32}$$

This embedding provides a close and discriminative representation of anxiety-related text features.

#### 3.2.2. Module 2: Depression Text Encoder

The depression encoder reflects the architecture and operations of the anxiety encoder (Module 1), independently learning depression-specific latent features $z_{d}\in R^{32}$. This dual-stream approach allows the proposed MCoG-LDPSNet model to disentangle anxiety- and depression-related signals effectively.

#### 3.2.3. Fusion and Loss-Driven Parametric Swish (LDPS)

Anxiety and depression embeddings are concatenated:

$$z_{f}=\left[z_{a};z_{d}\right]\in R^{64}$$

This joint representation is transformed and passed through the LDPS activation, which modulates the output gain via a learnable parameter β:

$$u=Dropout\;(ParametricSwish\;(W_{f}z_{f}+b_{f};\beta_{0});p=0.4)$$

The gain parameter β is constrained and dynamically modulated as follows:

$$\beta =clip\;\left(exp\left(log\beta \right),\epsilon ,\beta_{\max}=5\right),$$

with maximum gain clipped to 5 to prevent numerical instabilities during training.

The output is then computed by a gain-adaptive *hardsigmoid* gating:

$$v=u\cdot hardsigmoid\;\left(\beta \;u\right),v\in R^{32}$$

The trainable logβ parameter is optimized via a composite loss function:

$$L=L_{focal}+\lambda L_{brier},$$

- $L_{focal}\;$ addresses class imbalance by emphasizing complex examples (phasic gain adaptation);
- $L_{brier}\;$ serves as a calibration regularizer to constrain β (tonic gain restraint).

The respective gradients concerning logβ induce a biphasic modulation analogous to neuromodulatory gain control:

$\frac{\partial L_{focal}}{\partial log\beta }$, drives rapid β boosts, promoting swift, transient gain increases on hard examples.

$\frac{\partial L_{brier}}{\partial log\beta }$, applies slower β restraint persistent gain moderation to maintain calibration.

This biphasic adaptation of neuromodulatory mechanisms in biological neural systems has motivated the design of networks to dynamically handle feature amplification during training. It is worth noting that this is the first-of-its-kind LDPS activation to embed gain adaptation directly in the network—phasic–tonic modulation driven end-to-end by Focal Loss and Brier, inspired by neurobiological neuromodulation. To our knowledge, this is the first implementation of an activation function embedding end-to-end gain adaptation driven by dual phasic–tonic losses motivated by biological neuromodulation mechanisms.

#### 3.2.4. Output Layer

The classification output is obtained through a sigmoid activation applied to the LDPS-activated features:

$$\hat{y}=\sigma (w_{o}^{⊤}v+b_{o})$$

This design obviates the need for additional post-hoc calibration, yielding outputs that are inherently calibrated and robust to class imbalance.

#### 3.2.5. Transfer Learning Procedure

This research utilizes transfer learning to fine-tune the proposed MCoG-LDPSNet model, employing a Facebook dataset that contains indicators of anxiety and depression [28]. This approach leverages broader linguistic representations that MCoG-LDPSNet was trained on using large-scale data while adapting to domain-specific symptomatology of anxiety and depression, resulting in a robust MCoG-LDPSNet model capable of nuanced mental health prediction for anxiety and depression.

Parameters for anxiety and depression encoders are pre-trained on a large source dataset (Kaggle) by minimizing the total loss:

$$\theta_{anx}^{*}=arg\;min_{\theta_{anx}}L_{T}\left(f_{anx}\left(x;\theta_{anx}\right),y_{T}\right)$$

$$\theta_{dep}^{*}=arg\;min_{\theta_{dep}}L_{T}\left(f_{dep}\left(x;\theta_{dep}\right),y_{T}\right)$$

where $f_{dep}$ and $f_{dep}$ denote the anxiety and depression encoder modular networks, respectively, and $L_{T}$*T* is the combined loss on the source dataset T.

Transfer learning is performed by initializing parameters with pretrained weights and fine-tuning higher-level layers:

$$\theta_{anx}^{0}=\theta_{anx}^{*},\;\;\;\;\;\theta_{dep}^{0}=\theta_{dep}^{*}$$

During fine tuning, embedding layers are frozen to preserve learned representations, while higher-level layers, including orthogonal GRU modules, dropout, and dense layers, are trainable:

$$\theta_{T}=\left\{\theta_{frozen},\theta_{trainable}\right\}$$

$$\theta_{frozen}=\left\{\theta_{anx}^{*},\theta_{dep}^{*}\right\}$$

The fine-tuning objective remains the same:

$$\theta_{anx}^{*}=arg\;min_{\theta_{anx}}L_{T}\left(f_{anx}\left(x;\theta_{anx}\right),y_{T}\right)$$

$$\theta_{dep}^{*}=arg\;min_{\theta_{dep}}L_{T}\left(f_{dep}\left(x;\theta_{dep}\right),y_{T}\right)$$

The final prediction for a new input is computed by concatenating the anxiety and depression embeddings:

$$z=Concatenate\left(f_{anx}\left(x;\theta_{anx}^{*}\right),f_{dep}\left(x;\theta_{dep}^{*}\right)\right)$$

which is passed to the output layer classifier

$$\hat{y}=g\left(z;\theta_{out}^{*}\right)$$

where the output layer parameters with mean 0 and variance $\sigma^{2}$ are initialized as follows:

$$\theta_{out}^{0}∼N\left(0,\sigma^{2}\right)$$

and optimized according to the following:

$$\theta_{out}^{*}=arg\;mi{n_{\theta }}_{out}L_{T}\left(g\left(z;\theta_{out}\right),y_{T}\right)$$

where $\theta_{out}^{*}$ is learned by minimizing the loss on target data.

#### 3.2.6. Hyperparameters in the Proposed MCoG-LDPSNet

The architecture and hyperparameters for the proposed MCoG-LDPSNet model, despite its inspiration by the MCoRNNMCD-ANN, were carefully selected based on a combination of solid theoretical foundations and hands-on experimentation applied in the fields of NLP and deep learning, particularly in mental health prediction applications. This study employed a train, validation, and test split with percentages of 60%, 20%, and 20%, respectively.

- Embeddings: An embedding size of 64 was chosen to balance the trade-off between semantic expressiveness and model complexity. Prior works have exhibited that neural embeddings effectively capture semantic relationships while minimizing overfitting [63,64].
- SpatialDropout: A dropout rate of 0.2 was applied to the embedding outputs to regularize the model early in the feature extraction pipeline. SpatialDropout prevents the co-adaptation of embedding features across sequence timesteps, thereby improving generalization, as demonstrated in sequence modelling [65].
- Orthogonal GRU Units: The recurrent orthogonal units in the GRU were set to 32 to adequately capture temporal dependencies in the input sequence without introducing excessive model complexity. This choice was informed by both the sequence length (T = 100) and the dataset size, with experiments showing that larger GRU sizes resulted in marginal gains but increased the risk of overfitting [66].
- Dropout: Dropout rates of 0.3 in the dense layers and 0.4 in the fusion layers were chosen to mitigate overfitting in higher-level representations. These rates are consistent with standard dropout values in neural architectures for text classification tasks, and empirical tuning confirmed their effectiveness in stabilizing training and enhancing generalization [67].
- LDPS Gain Parameters: The LDPS activation parameters were initialized to moderate gain values to ensure stable gradient flow and model convergence. The maximum gain was clipped to 5 with a small epsilon of 10^−3^ to prevent numerical instabilities during training. To address class imbalance and calibration simultaneously, this study employed a Focal Loss, which emphasizes hard and minority examples, in conjunction with the Brier score, which encourages calibrated probability outputs [68].
- Batch Size and Learning Rate: To provide a stable and efficient training process, a learning rate of 0.001 and a batch of 128 were empirically determined, consistent with best practices in deep learning for moderate-sized datasets [69].

## 4. Results and Discussion

### 4.1. Technical Specifications and Performance Evaluation

To compare the proposed MCoG-LDPSNet with the benchmark computational models, objective evaluation classification metrics such as accuracy, F1 score, precision, recall, and sensitivity, as well as AUROC and G-mean, were considered the best metrics for the classification task. Moreover, the selected baseline models were chosen to be compared with the proposed MCoG-LDPSNet based on the limitations of Section 1, filling the future research in Section 2, which introduced a variety of machine learning algorithms like GLM, LR, SVM, NB, DT, RF, AdaBoost, and XGBoost applied in mental health predictions. Likewise, these models are used in Section 2, along with CNNs, LSTMs, BERT, and the state-of-the-art DeprMVM and CNN-BiLSTM-ATTN (CBA). The data from Kaggle was also used to train, test, and validate the sets for 19 classification algorithms, specifically for anxiety (class 0) and depression (class 1). All the computational models in the comparisons were used with the same portion for all the models, meaning 60%, 20%, and 20%. Moreover, the benchmarks utilized their parameters in studies applied to classification tasks related to mental health. Each model was trained and evaluated 50 times with distinct random seeds under Intel^®^ Core™ i7-9750H (Hyper-Threading Technology), 16 GB RAM, 512 GB PCIe SSD, NVIDIA GeForce RTX 2070 8 GB. The Anaconda computational environment, which includes Keras and TensorFlow, was utilized in Python (version 3.11) to conduct the experiments. Table 1 presents the results applied to the Kaggle dataset using seven performance metrics: accuracy, F1, precision, recall, specificity, geometric mean (G-mean), and area under the ROC curve (AUC), along with the running time of 50 times. Table 2 presents the standard deviation of the algorithms.

As shown in Table 1, the proposed MCoG-LDPSNet exhibited the best objective classification metrics, including G-mean and ROC analysis. The MCoG-LDPSNet was also compared and outperformed the MCoRNNMCD-ANN. However, it is worth noting that the MCoRNNMCD-ANN was the second-best performing model among the remaining ones. GRU showed that it can provide notably accurate predictions. At the same time, LSTM demonstrated its ability to retain information over extended periods and analyse complex data patterns, as evidenced by its improved metric outcomes. The state-of-the-art CNN-BiLSTM-ATTN (CBA) improved its classification metrics, demonstrating a sufficient balance between precision and recall. These models are effective due to their high AUC and G-mean scores.

Transformers like BERT performed substantially more poorly on AUC and G-mean with a tremendous runtime, which highlights both the difficulty of the task and the practical constraints around using transformers for low-latency mobile screening. At the same time, the simpler algorithms, such as Naïve Bayes models (multinomial and Gaussian), demonstrated significantly lower processing times but yielded far lower performance, making them the least promising option. Consequently, the proposed MCoG-LDPSNet outperformed the Bernoulli NB, which presented a lower AUC of 83.3% compared to MCoG-LDPSNet. In the G-mean, the SVM showed 287% lower performance than MCoG-LDPSNet.

The top-performing five models strike a successful balance between precision and recall, often utilizing advanced neural network structures to manage complex data. Table 3 provides a more in-depth comparison of the top-performing models.

Table 3 demonstrates that the proposed MCoG-LDPSNet model outperforms the top computational approaches. MCoG-LDPSNet achieved a superior AUC of 0.9920 and a balanced G-mean of 0.9451, with a runtime of 1237 s. These objective metrics set a high benchmark for evaluating the classification of real-world computational architectures.

The proposed MCoG-LDPSNet outperformed the MCoRNNMCD-ANN model, which showed a 0.49% lower AUC and a 1.76% lower G-mean than the MCoG-LDPSNet model. The MCoRNNMCD-ANN runtime was 86% slower than the MCoG-LDPSNet.

Similarly, the proposed MCoG-LDPSNet outperformed the CNN-BiLSTM-ATTN (CBA) model, which indicated a 0.92% lower AUC and a 2.35% lower G-mean than the MCoG-LDPSNet. Compared to runtime, it was 45% slower than MCoG-LDPSNet, making it less attractive for applications where processing time is critical.

MCoG-LDPSNet outperformed the GRU, which exhibited a 1.27% lower AUC and a 2.70% lower G-mean than MCoG-LDPSNet. GRU runtime was also 95% slower than MCoG-LDPSNet.

While offering reasonable performance, the LSTM model falls short compared to the proposed MCoG-LDPSNet model. LSTM records an AUC of 3.18% lower and a G-mean of 7.01% lower than the MCoG-LDPSNet. LSTM runtime is slower by 105% than MCoG-LDPSNet, suggesting that LSTM may be less suitable for time-sensitive tasks.

**Figure 3:**
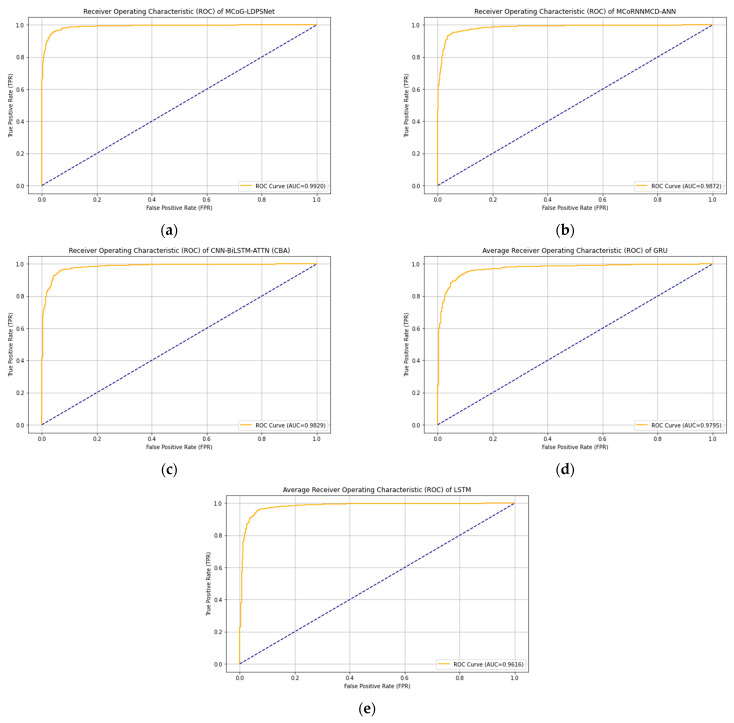
ROC of: (**a**) proposed MCoG-LDPSNet; (**b**) MCoRNNMCD-ANN; (**c**) CNN-BiLSTM-ATTN (CBA); (**d**) GRU; and (**e**) LSTM.

**Figure 4:**
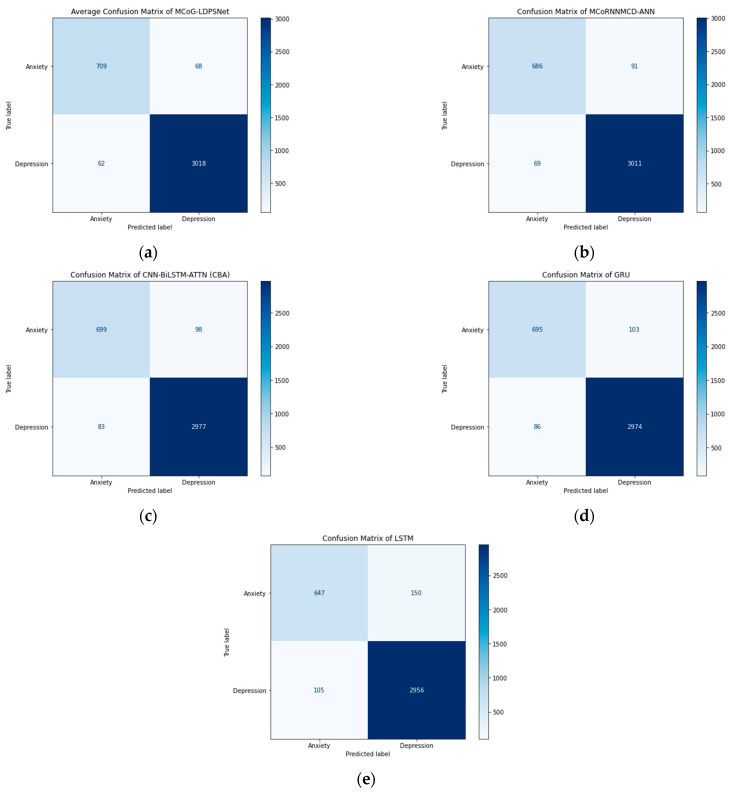
Confusion matrix of: (**a**) proposed MCoG-LDPSNet; (**b**) MCoRNNMCD-ANN; (**c**) CNN-BiLSTM-ATTN (CBA); (**d**) GRU; and (**e**) LSTM.

In summary, the proposed MCoG-LDPSNet remains the best overall model. The MCoRNNMCD-ANN, CNN-BiLSTM-ATTN (CBA), GRU, and LSTM models underperform the proposed MCoG-LDPSNet in classification objective metrics and suffer from significantly longer processing times. MCoG-LDPSNet has also improved the computational running time compared to MCoRNNMCD-ANN, showing not only its predictive power in imbalanced data but also a tremendous improvement in running time. These findings indicated the exceptional performance of the proposed MCoG-LDPSNet, which strikes a balance between predictive and computational efficiency, making it well-suited for the essentials of mental health prognosis, especially in the context of anxiety and depression. Finally, as shown in Table 2, MCoG-LDPSNet’s very low σ (AUC ± 0.0010, G-mean ± 0.0100) demonstrates exceptional consistency compared to the rest. Figure 3 illustrates the ROC of the top five models. Likewise, Figure 4 displays the confusion matrix of the top five approaches where the proposed MCoG-LDPSNet model shows an improvement over the MCoRNNMCD-ANN model. It correctly identifies more anxiety cases (709 vs. 686), and fewer anxiety cases are misclassified as depression (68 vs. 91). Similarly, it misclassifies fewer depression cases as anxiety (62 vs. 69) and correctly identifies more depression cases (3018 vs. 3011), suggesting better overall performance. The LSTM model performed the least effectively, particularly in correctly identifying cases of anxiety.

### 4.2. Transfer Learning

The outstanding performance of the proposed MCoG-LDPSNet has been fine-tuned in a domain-specific task with smaller data [28]. Transfer learning was crucial in our workflow because it enabled MCoG-LDPSNet to leverage rich, generalizable representations learned from a large source corpus, capturing diverse linguistic and emotional patterns before being fine-tuned on a smaller, domain-specific dataset for anxiety and depression. By freezing the lower-level embedding parameters and only updating the higher-order convolutional, recurrent, and LDPS layers, the model retained its robust feature extractors while adapting efficiently to new, scarce data. This strategy mitigated overfitting, accelerated convergence, and yielded significantly better performance in low-resource screening scenarios compared to training from scratch.

**Figure 5:**
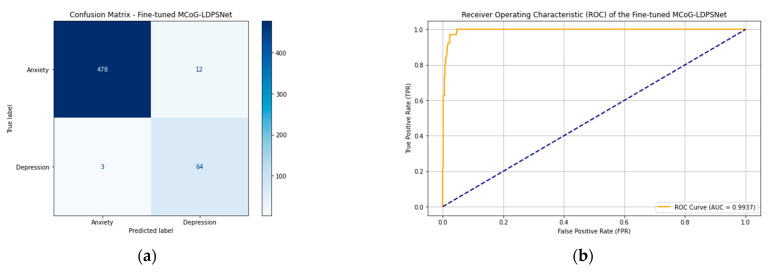
(**a**) Confusion matrix of the fine-tuned MCoG-LDPSNet; (**b**) ROC of the fine-tuned MCoG-LDPSNet.

Transfer learning achieved highly competitive performance across all evaluated metrics. With an average accuracy of 97.31% ± 0.23%, a precision of 99.29% ± 0.12%, and an F1 score of 98.46% ± 0.13%, the system demonstrated a strong discriminative capability in distinguishing between anxiety and depression narratives. Moreover, high recall (97.65%) and specificity (97.59%) values indicate that the model maintains a balanced capacity to catch both true positives and true negatives, which is critical in sensitive mental health screening contexts. The AUROC further confirms this robustness, averaging 0.9937 ± 0.0004, indicating near-perfect separability between the two emotional states. From a mental informatics perspective, these findings highlight the utility of NLP-driven approaches in early screening for affective disorders, particularly when combined with transfer learning. Figure 5 illustrates the confusion matrix, showing very few misclassifications between the two classes, along with the ROC curve of the fine-tuned MCoG-LDPSNet.

## 5. Practical Implications of the Proposed MCoG-LDPSNet

This analysis conducted a cohort study for evaluation of the real-world impact of integrating the MCoG-LDPSNet model into the EmotiZen App over 12 weeks, from 1 January to 31 March 2025. All data were collected during routine app usage and fully de-identified upon extraction. Only data from EmotiZen users who provided consent were included in this study, and all data were handled in accordance with the principles outlined in the Declaration of Helsinki. Users had previously agreed to the fully anonymized research use of their in-app responses via EmotiZen’s terms of service, rendering this minimal risk research exempt from additional institutional review. Due to the nature of this study and its alignment with GDPR standards, no further ethical approval was required.

### 5.1. Participants Identification and Screening

To validate the EmotiZen App and the proposed model’s realism in early screening of anxiety and depression, all eligible users were asked at study entry (January 2025) to complete both of the following:

- The standard multiple-choice PHQ-4 (MC PHQ-4; fixed-response, 0–3 per item);
- The app’s free-text PHQ-4 (open-ended, natural language).

These assessments were completed within the same onboarding window (the first study week), allowing for a direct comparison of the model’s mapped scores with the standard PHQ-4.

Moreover, the goal was to determine whether the proposed algorithm’s early predictions, integrated into the app, combined with the new features of the EmotiZen App, would enhance their mental well-being. This study observed two groups:

- Group A: Standard EmotiZen experience (anxiety and depression predictions, visualizations and iCBT recommendations by severity);
- Group B: Enhanced experience (anxiety and depression predictions, visualizations, and iCBT recommendations by severity and engagement features like progress and the (i) weekly push notifications, (ii) the option to choose their recommendation in a favourable order, (iii) a progress bar to track their advancements, and (iv) an incremental reward screening).

The cohort selection was made based on demographic metadata (age, gender, and region tag) and applied the following criteria:

Inclusion (January 2025)

- Age ≥ 18 years (based on self-reported birth year metadata);
- Residence in Hessen (Wiesbaden) or Rhineland-Palatinate (Mainz);
- Prior EmotiZen use before January 1 2025 (inferred from any login event in December 2024 or earlier);
- Proficiency in English (confirmed by the in-app language set to English);
- Total modified PHQ-4 score between 3 and 8 (mild to moderate) on any January submission.

Exclusion (January 2025)

- Total modified PHQ-4 ≥ 9 (“severe”);
- Any free-text response containing self-harm keywords (flagged by the NLP pipeline);
- Self-reported ongoing inpatient psychiatric care or recent hospitalization (<30 days);
- Language preference not set to English;
- No recorded login from a smartphone or computer during January 2025 (indicating unreliable access).

Of 82 users completing a January PHQ-4, 25 were excluded (12 for severe scores, 5 for self-harm risk, 3 for intensive treatment, and 5 for language/access). The remaining 57 users comprised the baseline cohort. By 31 March 2025, seven had not completed any Week 12 PHQ-4 and were excluded from the primary paired analysis, yielding a final analytic sample of 50 users.

### 5.2. EmotiZen App Components

Modified PHQ-4 Screening

Instead of fixed multiple-choice items, EmotiZen presents the four PHQ-4 questions as open-ended prompts, allowing users to express nuances in natural language. Each response is scored 0–3 for anxiety and 0–3 for depression (total 0–12). Users responded to four open-ended prompts corresponding to standard PHQ-4 items, each answered in free text:

- “Over the past two weeks, how often have you felt relaxed versus nervous, anxious, or on edge?”;
- “Over the past two weeks, how often have you felt you could stop worrying or control your worries?”;
- “Over the past two weeks, how often have you felt optimistic versus depressed or hopeless?”;
- “Over the past two weeks, how often have you felt engaged and motivated versus having little interest?”.

Each response was analyzed using the Affin sentiment lexicon, which assigns a numerical sentiment score to words and phrases [70]. The Affin-derived feature vectors for each PHQ-4 item were input into the proposed MCoG-LDPSNet model, which was initially trained for predicting and classifying anxiety and depression, yielding significant results, as shown in Section 4. The output probabilities of the MCoG-LDPSNet model are rule-based and mapped using fixed thresholds for four scores (0–3 each), which are summed to yield a total PHQ-4 score (0–12). The total PHQ-4 score is mapped to standard severity bands:

- None: 0–2;
- Mild: 3–5;
- Moderate: 6–8;
- Severe: 9–12.

After the MCoG-LDPSNet prediction and PHQ-4 mapping (model outputs converted to item scores using pre-specified probability thresholds), the EmotiZen App assigns the user to a severity band, suggesting one text-based iCBT task per week within that band. Tasks are prioritized and selected from a fixed, expert-defined hierarchy; an ordered clinician-ranked list matched to MCoG-LDPSNet outputs. The app filters the hierarchy for tasks eligible for the user based on symptom-target matching to the user’s item-level profile and current severity, then selects the highest-priority weekly task. For example, for “Mild,” breathing exercises may be recommended. EmotiZen offers text-based iCBT recommendations based on their potential effectiveness in improving mental well-being [59,60,61].

### 5.3. Timeline and Follow-Up

Baseline (Weeks 1–4, January):

- Both standard PHQ-4 and EmotiZen App questions were completed during onboarding.
- Demographics recorded.

Intervention (Weeks 5–8, February):

- Cohort A received screening for anxiety and depression and weekly iCBT delivery.
- Cohort B received the engagement (i) weekly push notifications, (ii) the option to choose their recommendation in a favourable order, (iii) a progress bar to track their advancements, and (iv) an incremental reward screening.

Follow-Up (Weeks 9–12, March):

- Continued weekly iCBT and PHQ-4, final Week 12 PHQ-4 endpoint, Week 12 satisfaction survey.
- Fifty users were followed for the whole 12-week period.

### 5.4. Statistical Validation and Interpretation

To test differences between groups, the *t*-test is appropriate, as it compares mean scores of approximately normally distributed variables from two independent groups. Within each arm, paired *t*-tests can be used to assess whether the pre- to post-feature changes are statistically significant. To quantify the magnitude of effects, Cohen’s d should be calculated for each *t*-test. Table 4 shows the participant demographics per group.

As observed in Table 4, the mean age difference is slight (33.6 vs. 35.2 years), and gender distributions are nearly identical (Group A: 60% F; Group B: 64% F). Matching on education and employment was also confirmed, ensuring that any downstream effects are unlikely driven by baseline demographic imbalances.

Moreover, Table 5 shows the outcomes of task performance for each group.

Based on Table 5, we observed that Group B showed a larger total drop (1.08 points) than Group A (0.80 points). Subscale improvements mirror this: anxiety decreased by 0.44 points versus 0.20 points, and depression decreased by 0.64 points versus 0.60 points. Task completion was substantially higher in Group B (85% vs. 65%), suggesting that greater engagement with deep learning-ranked recommendations contributed to the additional 0.28-point improvement on the NLP (PHQ-4).

The post-features between group comparisons are illustrated in Table 6.

Table 6 shows that differences between-groups were conducted with independent-samples *t*-tests $df=n_{1}+n_{2}-2$ are statistically significant at week-12: anxiety t(48) = 2.48, *p* = 0.017 (Cohen’s d = −0.70, medium-large), depression t(48) = 3.38, *p* = 0.001 (Cohen’s d = −0.96, large), and total PHQ-4 t(48) = 6.35, *p* < 0.001 (Cohen’s d = −1.80, very large). Eventually, Group B had lower (better) week-12 scores. These results indicate that the enhanced engagement features in Group B were associated with clinically meaningful and statistically significant greater symptom reductions compared with the standard app features in this cohort. The final improvements in outcomes are presented in Table 7.

Group B’s total drop of 1.08 points exceeds Group A’s 0.80 by 0.28 points. Anxiety improvement is more than double (0.44 vs. 0.20), while depression gains are marginally higher (0.64 vs. 0.60). These numbers support the data-driven, optimized engagement which produces more robust symptom relief across domains. The NLP (PHQ-4) is a set of questions that users answer via the EmotiZen App.

**Figure 6:**
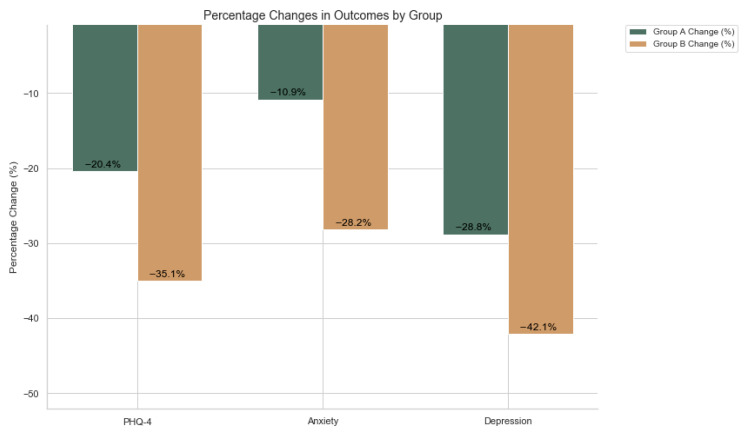
The changes in outputs per group.

Finally, Figure 6 illustrates the mean percentage reductions in PHQ-4 total and subscale scores from baseline to week 12 for Groups A and B, calculated from the group means in Table 5. Group A’s PHQ-4 total score decreased from 3.92 to 3.12, an absolute change of 0.80, representing a 20.4% reduction, while Group B’s score declined from 3.08 to 2.00, an absolute change of 1.08, corresponding to a 35.1% reduction. For the anxiety subscale, Group A’s score dropped from 1.84 to 1.64 (Δ = 0.20, −10.9%), whereas Group B’s declined from 1.56 to 1.12 (Δ = 0.44, −28.2%). On the depression subscale, Group A showed a decrease from 2.08 to 1.48 (Δ = 0.60, −28.8%), and Group B from 1.52 to 0.88 (Δ = 0.64, −42.1%). Across all measures, Group B demonstrated consistently greater relative reductions in symptoms than Group A, indicating a more substantial improvement in both anxiety and depression over the 12 weeks.

### 5.5. Post-Hoc Correlation Analysis of the Proposed MCoG-LDPSNet Predictions vs. Mental Health Professional-Administered PHQ-4

To further validate the proposed MCoG-LDPSNet predictive performance deployed in EmotiZen App screening, we conducted Pearson correlations between model-predicted subscale/total scores (via the app) and contemporaneous mental health expert-administered PHQ-4 scores across all participants and within each study. Table 8 illustrates the correlations between the proposed MCoG-LDPSNet predictions and standard PHQ-4.

The correlations in Table 8 were high (total r ≈ 0.974; anxiety r ≈ 0.976; depression r reported as near 1.000). These high r values indicate strong face validity of the mapping in this cohort analysis. These results (Table 8) provide compelling face validity for the proposed MCoG-LDPSNet algorithm. The proposed model’s almost perfect concordance with gold-standard mental health expert ratings suggests that the following:

- Semantic fidelity: Open-ended responses retain the same severity information as fixed-choice items when processed through the proposed MCoG-LDPSNet architecture.
- Clinical interchangeability: In routine use, EmotiZen’s AI-driven scores can reliably substitute for in-person PHQ-4 administration, enabling more scalable and user-friendly screening.
- Robustness to engagement differences: high correlations in both cohorts confirm that extra app features do not distort the model’s predictive accuracy.

Combined with the primary analyses showing significant symptom reductions correlated with app engagement, this post-hoc correlation underscores the dual utility of EmotiZen as an accurate digital screener capable of improving mental health well-being.

**Figure 7:**
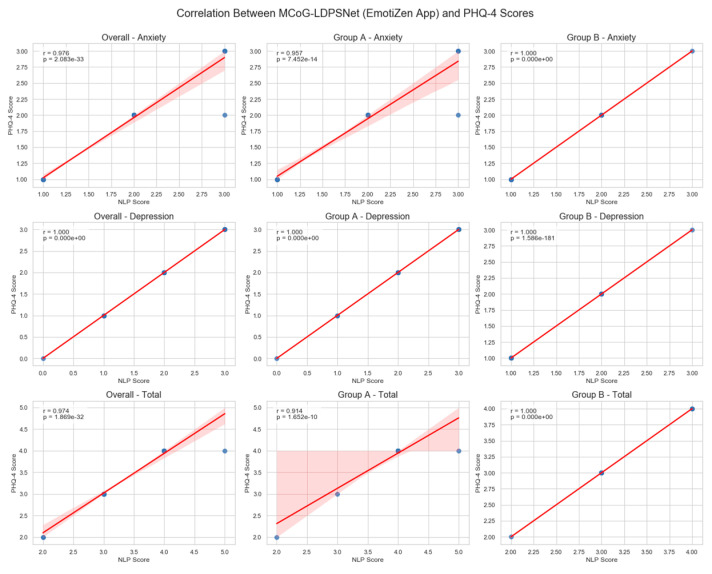
Scatterplots and linear fits of NLP-predicted vs. clinician-administered PHQ-4.

Figure 7 shows the near-unity correlation values across all panels, confirming that the proposed MCoG-LDPSNet algorithm replicates mental health PHQ-4 assessments with exceptional fidelity. This strong evidence supports the deployment of EmotiZen’s free-text screening as a valid proxy for the traditional PHQ-4 in both research and real-world mental health workflows.

Following the results presented in Section 4 and Section 5, both of our guiding questions are conclusively addressed:

- Detection Efficacy: MCoG-LDPSNet demonstrated a marked improvement in identifying anxiety and depression under extreme class imbalance, achieving a 4.5% increase in AUROC and a 7.01% gain in G-mean over leading benchmarks, including GLM, XGBoost, DeprMVM, CNN-BiLSTM-ATTN, and BERT. These gains confirm that our Loss-Driven Parametric Swish activation and adaptive gain control mechanism substantially enhance sensitivity to minority-class patterns without sacrificing overall calibration or robustness.
- Mobile Feasibility: When embedded in the EmotiZen App and fine-tuned via transfer learning on social media user data, MCoG-LDPSNet not only sustained its predictive accuracy in a live setting but also scaled seamlessly across diverse user profiles. The enhanced version of EmotiZen, which leverages on-demand screening, personalized iCBT recommendations, and engagement tools, yielded higher task completion rates (85% vs. 65%) and greater symptom reduction (1.08 vs. 0.80 total NLP-PHQ-4 points). These developments underscore that incorporating the proposed MCoG-LDPSNet into the EmotiZen App significantly improves the real-time identification of anxiety and depression, personalization, and user engagement, thereby validating its practical utility for scalable, frontline mental health support.

### 5.6. Ethical Considerations

This study was designed with the principle that technology should serve to augment, not supplant, human judgment and care. By embedding the proposed MCoG-LDPSNet model within the EmotiZen App, we ensured that all predictions of anxiety and depression severity remained transparent and interpretable to both users and clinicians. At every step, users retained control over their data and the subsequent iCBT recommendations: They could review, modify, or override the app’s suggestions and were free to opt in or opt out of any feature. To ensure that users do not rely exclusively on the algorithm without also receiving guidance on mental health, automated sentiment scoring and thresholding were combined with concise instructions on how to use the app and links to expert resources. This approach emphasizes the collaborative use of intelligence and user engagement to improve human understanding while preserving user autonomy and privacy.

## 6. Conclusions

This work introduces the MCoG-LDPSNet, a novel variation of MCoRNNMCD-ANN and a brain-inspired, adaptive-gain architecture specifically designed to overcome class-imbalance pitfalls that plague existing GLM, XGBoost, DeprMVM, CNN-BiLSTM-ATTN, and transformer-based methods applied to many mobile apps and platforms. By integrating a novel, learnable β-parameter in the Loss-Driven Parametric Swish layer, calibrated through confidence-aware loss signals, MCoG-LDPSNet dynamically reshaped its activation to enhance minority-class sensitivity.

In rigorous head-to-head benchmarks, the proposed MCoG-LDPSNet achieved an impressive 83.3% AUC gain against models such as Bernoulli NB, and a G-mean outstanding improvement of 287% against SVM. Transformers like BERT performed substantially poorly on these metrics with an extensive runtime of 630,400 s, which highlights both the hardship of the task and the practical constraints around using transformers for low-latency mobile screening. Furthermore, this study did not utilize larger pre-trained models on heterogeneous web-scale corpora whose content, biases, and license conditions are often opaque; relying on such weights can introduce unknown representational priors and governance complications, which are especially important in sensitive mental health applications. Against the top five models, the proposed MCoG-LDPSNet outperformed them, including the second-best model of this study, which was our previous MCoRNNMCD-ANN, by 0.49% in AUC and 1.76% in G-mean. Notably, the MCoG-LDPSNet runtime was 86% faster than the MCoRNNMCD-ANN, showing not only an improvement in performance but also a tremendous speed improvement, making it more sustainable and cost-effective in computational time.

When deployed in the EmotiZen App and fine-tuned via transfer learning on real-world user data, the proposed MCoG-LDPSNet model delivered highly reliable screening, resulting in meaningful improvements in user engagement and symptom reduction. These results demonstrate that MCoG-LDPSNet not only pushes the boundaries of deep learning for mental health in terms of technology but also has excellent potential for more scalable, on-demand, and equitable screening, which would enable earlier intervention and better outcomes for various populations.

However, limitations must be acknowledged. First, the sample during the cohort analysis was relatively small (*n* = 50) and geographically constrained to two German states, which may limit generalizability. Second, reliance on self-reported, free-text responses introduces potential biases (e.g., social desirability) that may affect the model’s mapping accuracy. Future research should involve larger, more diverse cohorts and randomized controlled trials to confirm efficacy across different cultures and languages. Extending this study, we also envision adaptive interventions that are dynamically tailored not only to anxiety and depression but also to other diseases of the central nervous system, like multiple sclerosis. Ultimately, integrating behavioural data, such as activity patterns, could enrich the model’s context awareness, enabling personalized digital mental health care that empowers humanity.

## Figures and Tables

**Table 1:** Mean of objective evaluation metrics.

Model	Accuracy	F1	Precision	Recall	Specificity	G-Mean	AUC	Time for 50 Runs (s)
Logistic Regression	0.8028	0.8878	0.8133	0.9773	0.1113	0.3295	0.6818	5.60
Multinomial NB	0.6704	0.7747	0.8525	0.7101	0.5132	0.6035	0.6301	1.27
Bernoulli NB	0.6609	0.7790	0.8118	0.7488	0.3123	0.4835	0.5411	1.68
KNN	0.7867	0.8774	0.8109	0.9558	0.1166	0.3335	0.5965	92.47
SVM	0.8076	0.8921	0.8077	0.9963	0.0600	0.2441	0.7232	5200.28
AdaBoost	0.8246	0.8957	0.8530	0.9429	0.3558	0.5790	0.8296	196.60
XGBoost	0.8560	0.9147	0.8676	0.9673	0.4152	0.6336	0.8787	114.84
CatBoost	0.8379	0.9052	0.8491	0.9692	0.3177	0.5548	0.8512	95.54
GLM	0.8027	0.8878	0.8133	0.9773	0.1113	0.3295	0.6818	40.74
Decision Tree	0.8087	0.8886	0.8305	0.9558	0.2263	0.4627	0.7820	17.57
Random Forest	0.8369	0.9063	0.8369	0.9882	0.2371	0.4838	0.8569	402.46
GRU	0.9509	0.9691	0.9665	0.9717	0.8709	0.9199	0.9795	3470.50
LSTM	0.9338	0.9588	0.9530	0.9657	0.8113	0.8795	0.9616	3984.50
CNN	0.9051	0.9412	0.9259	0.9574	0.7048	0.8205	0.9482	1176.66
BERT	0.7670	0.8339	0.8845	0.8143	0.5411	0.5958	0.6730	630,400.00
DeprMVM	0.9008	0.8098	0.8238	0.7989	0.9378	0.8650	0.9091	111.50
CNN-BiLSTM-ATTN (CBA)	0.9529	0.9704	0.9681	0.9728	0.8765	0.9232	0.9829	1958.75
MCoRNNMCD-ANN (ours)	0.9583	0.9740	0.9706	0.9775	0.8825	0.9286	0.9872	3093.00
**MCoG-LDPSNet (ours)**	0.9660	0.9787	0.9778	0.9797	0.9119	**0.9451**	**0.9920**	1237.00

**Table 2:** Standard deviations.

Model	Accuracy	F1	Precision	Recall	Specificity	G-Mean	AUC
Logistic Regression	0.0058	0.0037	0.0059	0.0030	0.0098	0.0144	0.0101
Multinomial NB	0.0073	0.0060	0.0074	0.0082	0.0194	0.0115	0.0102
Bernoulli NB	0.0086	0.0067	0.0069	0.0088	0.0119	0.0103	0.0095
KNN	0.0064	0.0041	0.0064	0.0040	0.0110	0.0158	0.0104
SVM	0.0062	0.0038	0.0061	0.0014	0.0076	0.0155	0.0095
AdaBoost	0.0046	0.0030	0.0056	0.0040	0.0203	0.0160	0.0060
XGBoost	0.0055	0.0035	0.0056	0.0035	0.0173	0.0132	0.0062
CatBoost	0.0058	0.0036	0.0059	0.0038	0.0154	0.0135	0.0064
GLM	0.0058	0.0037	0.0059	0.0030	0.0098	0.0144	0.0101
Decision Tree	0.0054	0.0035	0.0088	0.0107	0.0442	0.0429	0.0082
Random Forest	0.0056	0.0035	0.0059	0.0022	0.0149	0.0152	0.0068
GRU	0.0078	0.0050	0.0044	0.0079	0.0175	0.0106	0.0053
LSTM	0.0286	0.0165	0.0328	0.0166	0.1509	0.0948	0.0246
CNN	0.0070	0.0041	0.0128	0.0120	0.0582	0.0303	0.0056
BERT	0.1230	0.0815	0.0671	0.1159	0.0178	0.0105	0.1852
DeprMVM	0.0184	0.0417	0.0491	0.0557	0.0174	0.0310	0.0329
CNN-BiLSTM-ATTN (CBA)	0.0029	0.0018	0.0073	0.0072	0.0299	0.0129	0.0015
MCoRNNMCD-ANN (our)	0.0035	0.0021	0.0074	0.0060	0.0313	0.0142	0.0019
**MCoG-LDPSNet (our)**	0.0039	0.0024	0.0053	0.0058	0.0220	**0.0100**	**0.0010**

**Table 3:** Top five models by AUC and G-mean.

Rank	Model	AUC	G-Mean
1	**MCoG-LDPSNet (ours)**	**0.9920**	**0.9451**
2	MCoRNNMCD-ANN (ours)	0.9872	0.9286
3	CNN-BiLSTM-ATTN (CBA)	0.9829	0.9232
4	GRU	0.9795	0.9199
5	LSTM	0.9616	0.8795

**Table 4:** Participant demographics (*n* = 25 per group).

Characteristic	Group A (Control)	Group B (Enhanced)
Participants	25	25
Mean Age (years)	33.6 ± 3.5	35.2 ± 2.0
Gender (F/M)	15/10	16/9
Education Level	Similar	Similar
Employment Status	Similar	Similar

**Table 5:** Outcomes and task performance.

Measure	Group A	Group B
Baseline PHQ-4	3.92 ± 0.49	3.08 ± 0.40
Post-PHQ-4	3.12 ± 0.67	2.00 ± 0.58
Absolute Improvement	0.80	1.08
Baseline Anxiety	1.84 ± 0.69	1.56 ± 0.58
Post-Anxiety	1.64 ± 0.81	1.12 ± 0.67
Anxiety Reduction	0.20	0.44
Baseline Depression	2.08 ± 0.76	1.52 ± 0.59
Post-Depression	1.48 ± 0.65	0.88 ± 0.60
Depression Reduction	0.60	0.64
Task Completion (%)	65.0 ± 12.5	85.0 ± 14.4

**Table 6:** Post intervention between group comparisons.

Measure	Group A	Group B	t(48)	*p*-Value	Cohen’s d
Anxiety	1.64 ± 0.81	1.12 ± 0.67	2.48	0.017	–0.70
Depression	1.48 ± 0.65	0.88 ± 0.60	3.38	0.001	–0.96
Total NLP (PHQ-4)	3.12 ± 0.67	2.00 ± 0.58	6.35	<0.001	–1.80

**Table 7:** Improvements in outcomes.

Measure	A: Initial	A: Post	A: Δ	B: Initial	B: Post	B: Δ
Anxiety	1.84	1.64	0.20	1.56	1.12	0.44
Depression	2.08	1.48	0.60	1.52	0.88	0.64
Total NLP (PHQ-4)	3.92	3.12	0.80	3.08	2.00	1.08

**Table 8:** Pearson correlations between MCoG-LDPSNet predictions and standard PHQ-4 scores.

Cohort	Measure	r	*p*-Value
Overall	Anxiety	0.976	2.1 × 10^−33^
	Depression	1.000	<1 × 10^−300^
	Total	0.974	1.9 × 10^−32^
Group A	Anxiety	0.957	7.5 × 10^−14^
	Depression	1.000	<1 × 10^−300^
	Total	0.914	1.7 × 10^−10^
Group B	Anxiety	1.000	<1 × 10^−300^
	Depression	1.000	1.6 × 10^−181^
	Total	1.000	<1 × 10^−300^

## Data Availability

The Kaggle mental health sentiment corpus (https://www.kaggle.com/code/mesutssmn/sentiment-analysis-for-mental-health/input, last accessed on 30 May 2025) and the Islam et al. dataset [28] are publicly accessible. By contrast, our 12-week EmotiZen cohort data cannot be shared due to participant anonymity and the sensitivity of health information, as mandated by applicable data protection regulations.
